# Immunobiology of Acute Chorioamnionitis

**DOI:** 10.3389/fimmu.2020.00649

**Published:** 2020-04-16

**Authors:** Monica Cappelletti, Pietro Presicce, Suhas G. Kallapur

**Affiliations:** Divisions of Neonatology and Developmental Biology, David Geffen School of Medicine at the University of California Los Angeles, Los Angeles, CA, United States

**Keywords:** inflammation, infection, immune cells, animal model, fetal membrane

## Abstract

Acute chorioamnionitis is characterized by neutrophilic infiltration and inflammation at the maternal fetal interface. It is a relatively common complication of pregnancy and can have devastating consequences including preterm labor, maternal infections, fetal infection/inflammation, fetal lung, brain, and gastrointestinal tract injury. In this review, we will discuss current understanding of the pathogenesis, immunobiology, and mechanisms of this condition. Most commonly, acute chorioamnionitis is a result of ascending infection with relatively low-virulence organisms such as the Ureaplasma species. Furthermore, recent vaginal microbiome studies suggest that there is a link between vaginal dysbiosis, vaginal inflammation, and ascending infection. Although less common, microorganisms invading the maternal-fetal interface via hematogenous route (e.g., Zika virus, Cytomegalovirus, and Listeria) can cause placental villitis and severe fetal inflammation and injury. We will provide an overview of the knowledge gleaned from different animal models of acute chorioamnionitis and the role of different immune cells in different maternal-fetal compartments. Lastly, we will discuss how infectious agents can break the maternal tolerance of fetal allograft during pregnancy and highlight the novel future therapeutic approaches.

## Introduction

Intrauterine infection or inflammation (IUI), also known as chorioamnionitis, is responsible for ~40% of preterm labor cases (1). Prematurity, which affects nearly 10% of pregnancies world-wide, is the most significant cause of perinatal mortality or morbidity (2).

In this paper, we explore the current knowledge of the mechanisms of IUI. In particular, we review how inflammation is propagated in different tissue compartments at the maternal-fetal interface, the role of resident cells interacting with immune cells at the interface, the role of inflammatory mediators, and how host-microbe interactions affect pathology. Although sterile inflammation (3, 4), environmental pollutants (5–7), cigarette smoke (8, 9), and other toxicants play an important role in the pathogenesis of IUI, these considerations are beyond the scope of this review.

Intraamniotic infection is an infection with resultant inflammation of any combination of the amniotic fluid (AF), placenta, fetus, fetal membranes, or decidua. The amniotic sac is composed of maternal (decidua) and fetal components (chorion and amniotic membranes) which surround the fetus and represent one site of maternal/fetal immune interaction. The amnion is a fetal tissue comprising a layer of epithelial cells and underlying mesenchymal cells, and an extracellular matrix and collagen that has a high tensile strength. The chorion is composed of a reticular layer, basement membrane, and trophoblasts. The decidua, the transformed maternal endometrium of pregnancy, is in direct proximity with the chorion and consists of maternal immune cells, decidual stromal cells, and extravillous fetal trophoblasts (10). Placental villous and intervillous space have a specialized architecture adapted for nutrient exchange and have distinct immune cells. The cellular interactions within these layers and in the placenta are important in coordinating the immune response for maintaining a semi-allogeneic fetus (11, 12).

Intraamniotic infection and chorioamnionitis are commonly used interchangeably; however, these two conditions are not the same, as we will discuss below. Furthermore, chorioamnionitis can be induced by sterile damage-associated molecules (13, 14).

### Clinical Definitions

Most often, the diagnosis of chorioamnionitis is made clinically based on the presence of fever, uterine tenderness, maternal leukocytosis, purulent cervical drainage, or fetal tachycardia (15). However, due to the vague nature of the definition and heterogeneity of clinical manifestations, an NIH expert panel proposed to replace the term “chorioamnionitis,” with a more general, descriptive term, “Intrauterine Inflammation and/or Infection,” abbreviated as *Triple I* (16). In this scheme, fever alone during labor is classified separately, while “suspected Triple I” is classified as fever with one or more of the following symptoms: leukocytosis, fetal tachycardia, or purulent cervical discharge. In order to be confirmed, “suspected Triple I" should be accompanied by AF infection (e.g., positive gram stain for bacteria, low AF glucose, high WBC count in the absence of a bloody tap, and/or positive AF culture results) or histopathological evidence of infection/inflammation in the placenta, fetal membranes or the umbilical cord vessels (funisitis).

### Anatomy of Fetal Membranes

There are four fetal membranes early in fetal life: the chorion, amnion, yolk sac, and allantois. The chorion and amnion are derived from trophoblastic ectoderm and extraembryonic somatic mesoderm. The yolk sac and allantois are derived from endoderm and extraembryonic splanchnic mesoderm. In humans, the yolk sac degenerates with fetal growth while the allantois is vestigial and may regress, but the blood vessels persist as umbilical arteries connect the embryo with the placenta (17). The reproductive tissues of mammals have many features in common but there are unique species-associated characteristics. For example, the development of fetal membranes in rodents is unique to those species and there are significant architectural differences between rodent and human placenta, although both have hemochorial placentation (18). Specifically, rodents have an inverted yolk sac placenta, where the fetal endoderm lies between the maternal tissue and the mesoderm, while in other species the fetal mesoderm lies between the ectoderm and endoderm (17, 19).

### Histopathological Definitions

**Figure 1:**
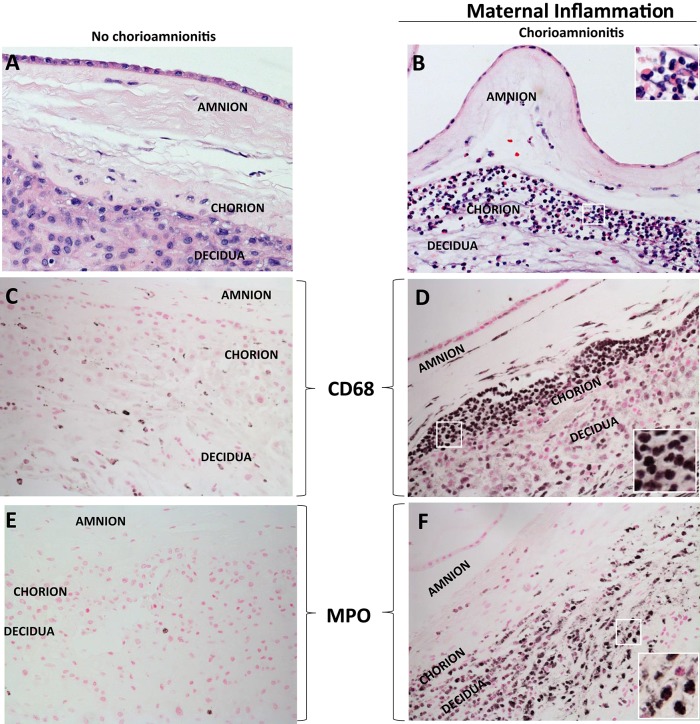
H&E histology of intrauterine inflammations. **(A,B)** Cross sections of human fetal membranes H&E histology showing neutrophil infiltration. Chorioamnionitis is characterized by infiltration of **(D)** CD68+ macrophages and **(F)** neutrophils expressing Myeloperoxidase+ (MPO) predominantly located at the choriodecidua junction. Note relatively much fewer CD68 or MPO expressing cells in the no chorioamnionitis group **(C,E)**. Insets in **(B,D,F)** show higher power magnification of demarcated area in white and demonstrate inflammatory cells including neutrophils and macrophages.

**Figure 2:**
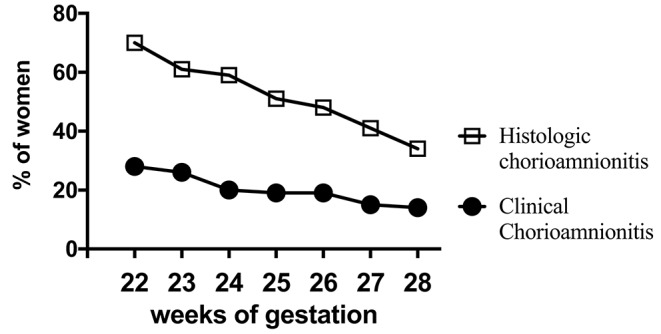
Chorioamnionitis during second trimester. Higher documentation of histologic vs. clinically diagnosed chorioamnionitis in the same mothers whose Infants were born at 22–28 weeks Gestational Age (GA) in the NICHD funded Neonatal Network database (2003-2007). Also note that chorioamnionitis is more frequently diagnosed at earlier gestations (inverse correlation of gestational age with incidence of chorioamnionitis). Adapted from Stoll et al. (23).

Acute inflammation characterized by the infiltration of neutrophils in the chorion and/or amnion is termed acute chorioamnionitis (1), and can involve the placental and/or extraplacental fetal membranes. “Maternal inflammation” refers to the infiltration of largely maternal neutrophils and macrophages in the fetal tissues of the chorion and amnion (Figure 1). Inflammatory processes involving the umbilical cord (umbilical vein, umbilical artery, and the Wharton's jelly) are referred to as acute funisitis, and constitutes fetal inflammation or fetal inflammatory response syndrome (FIRS). Placental inflammation affecting the villous tree is called acute villitis. A widely used classification by Redline (20) further divided the maternal and fetal inflammatory response into stages and grades. The term “stage” refers to the progression of the inflammatory process based on the anatomical regions infiltrated by neutrophils; the term “grade” refers to the intensity of the acute inflammatory process at a particular site. Interestingly, the characteristic location of initial neutrophil infiltration is the choriodecidual junction, with invasion into the amnion denoting higher stages of inflammation. The incidence of histologic chorioamnionitis is inversely related to the gestational age at preterm delivery (defined as delivery <37 weeks' gestation) (21). Interestingly, histologic chorioamnionitis is diagnosed in >70% of preterm births occurring at <28 weeks' gestation (22, 23) (Figure 2). The precise reasons for different rates of chorioamnionitis at different gestational ages are not clear. One possibility is gestational dependence of immune function (24). Studies have shown that the expression of innate immune receptors [e.g., Toll-like receptors (TLRs)] in the placenta (24, 25) and fetal membranes are increased after 20 weeks of pregnancy (26). The vast majority of preterm deliveries occur in the late third trimester with medically indicated preterm deliveries contributing to ~30% of cases (22). This may also decrease the proportion of prematurity attributable to infection/inflammation during the late third trimester.

### Microbiology

#### Ascending Infections

The most common route for microbes to cause intrauterine infection is by ascending from the lower genital tract (1). The most frequent microorganisms found in the amniotic cavity are genital mycoplasmas, in particular the *Ureaplasma* species (27). Of note, *Group B Streptococcus* (GBS) colonizes the genitourinary tract in 20–30% of pregnant women (28) and is associated with chorioamnionitis, preterm premature rupture of amniotic membrane (PPROM) and preterm birth (29–31). Other common organisms include *Gardenella vaginalis*, Fusobacteria species (32–35), and *E. coli* (36). Although not reported frequently, fungi can also cause chorioamnionitis. Among the fungi, Candida species, particularly *C. albicans* (37, 38) and less commonly *C. glabrata* (39, 40) have been reported. Unlike invasive infections in other parts of the body, polymicrobial invasion of the amniotic cavity is present in ~30% of cases (41).

#### Hematogenous Infections

In a minority of cases, microorganisms can also invade the placenta by the hematogenous route, and the profile of organisms is different compared to the ascending route. Microorganisms that invade the placenta by the hematogenous route include *Listeria monocytogenes* (42), *Zika virus* (43, 44), *Treponema pallidum* (45), Cytomegalovirus (46), Plasmodium species (47), and microorganisms causing toxoplasmosis, syphilis, varicella-zoster, parvovirus B19, Rubella, and Herpes infections (TORCH) (48). These organisms gain access through the maternal circulation to the intervillous space, from where they invade the villi and fetal circulation. The oral pathogen *Fusobacter nucleatum* can also cause IUI through the hematogenous route (49). Indeed, bad oral hygiene and multiple different oral pathogens have been implicated in IUI and preterm births, although a randomized controlled trial of treatment of periodontal disease did not reduce adverse pregnancy outcomes (50). In contrast to the ascending infections causing inflammation primarily in the choriodecidua and amnion, organisms invading through the hematogenous route cause inflammation primarily in the placental villi and intervillous space.

#### Influenza Infection

Pregnant women are at increased risk of developing severe disease with seasonal influenza, being hospitalized at a rate of 1–2/1,000, a risk that is 18-fold greater than that for healthy non-pregnant women (51–55). Severe maternal infections such as influenza—particularly pandemic infections (e.g., 1918 and 2009 pandemics and 2005 avian influenza), can lead to still birth and preterm delivery, although the precise mechanisms of the disease are not well-understood (56). Influenza vaccines are one of the most effective interventions. An epidemiological study suggests that vaccination against influenza virus protects from preterm birth (57, 58). Generally, viruses may impact maternal and fetal health by infecting gestational tissues and modulating intrauterine immune responses (59, 60). However, whether influenza virus infection can cause chorioamnionitis is not known.

#### Priming Infections

Viral infections, such as influenza virus, can prime or accentuate bacterial infection-mediated preterm labor and the intensity of inflammatory response at the maternal-fetal interface (61–66). Specifically, pathogen/pathogen-associated molecular pattern-driven activation of type I Interferon (IFN)/IFN receptor (IFNAR) was sufficient to prime for systemic and uterine proinflammatory chemokine and cytokine production and induction of preterm birth in mice (67). The synergy during combined stimulation of different Toll-like receptors (TLRs) is considered to be a “two-hit hypothesis” (68). These findings might explain how subclinical type I IFN-producing systemic infections (either virus or bacteria) act as initial inflammatory triggers and increase susceptibility for secondary inflammatory challenge-driven adverse pregnancy outcomes (67).

#### Vaginal Microbiome

Bacterial vaginosis (BV), a dysbiotic state of the vagina, is known to be a risk factor for prematurity (69). Recent metagenomic studies have shed light on normal composition of vaginal microorganisms during the non-pregnant state and during pregnancy in women of different racial groups. Compared to the gastrointestinal (GI) microbiome, the vaginal microbiota has much less alpha-diversity (different microbial species within the vaginal ecosystem) (70). Notably, the vaginal microbiota has a *Lactobacillus* predominance during pregnancy, different from the GI microbiota, but the vaginal microbiota becomes more similar to the GI microbiota during the postpartum period with less *Lactobacillus* predominance (70). Five different profiles of vaginal community states in non-pregnant women of reproductive age were described (71). Type I profile is dominated by *L. crispatus*, type II is dominated by *L. gasseri*, type III is dominated by *L. iners*, type IV-A or IV-B are characterized by high relative abundance of species of genus *Atopobium, Prevotella, Sneathia, Gardnerella, Ruminococcaceae, Parvimonas, Mobiluncus*, and other taxa previously shown to be associated with bacterial vaginosis. Type V is dominated by *L. jensenii* (71). Compared to vaginal microbiota of non-pregnant women, the vaginal microbiota during pregnancy tends to be relatively stable with a dominance of *Lactobacilli* species (72). Recent microbiota studies have clarified that vaginal *Lactobacillus* deficiency, particularly *L. crispatum* deficiency and dominance of community state type IV accompanied by elevated Gardnerella or Ureaplasma abundances (vaginal dysbiosis) predisposes to prematurity (70, 73). Furthermore, dysbiotic organisms have higher transcriptomic activity in women delivering preterm compared to those delivering at term (74). Lactobacillus predominance is important because it is protective for increased vaginal microbiological diversity, and growth of type IV organisms. Prematurity was associated with increased abundance of pro-inflammatory cytokines in vaginal wash (74), and dysbiotic vaginal flora was associated with preterm premature rupture of membranes (PPROM), chorioamnionitis, funisitis, and early onset neonatal sepsis (73). Thus, vaginal dysbiosis appears to cause local vaginal inflammation and increase the likelihood of an ascending infection and chorioamnionitis, leading to increased risk for prematurity. Studies in different racial groups have shown that there are significant racial variations in the vaginal microbiota. The association between the lower frequency of *Lactobacillus* and higher *Gardnerella* with increased risk for PTB was demonstrated in Caucasian but not African American women. While a lower abundance of *Lactobacillus crispatus* was associated with low risk of preterm birth in both cohorts, the presence of *Lactobacillus iners* was not associated with the risk for PTB (75). Thus, more work is needed to understand how vaginal flora may play a role in ascending infection and prematurity in different racial groups.

## Compartments Within the Intrauterine Space and Pathways of Microbial Invasion

The intrauterine space has several sub-compartments that although contiguous have distinct immune and functional roles. The sub-compartments include the amniotic fluid, fetus, choriodecidua, amnion, villous placenta, and the uterus. One particular cytokine, IL-6, was identified as a critical marker of intrauterine inflammation (76) and a predictor of preterm birth (77). Experimental evidence supports the crucial role of IL-6 during parturition. In mice, IL-6 deficiency delayed normal parturition (78), and protected from inflammation-driven preterm birth (67). Normally, intrauterine infection is mainly a localized inflammatory process. In humans, IL-6 levels are >100-fold higher in the amniotic fluid compared to the maternal or fetal blood during IUI (79, 80). This information is important in interpreting the relevance of animal models of intrauterine infection induced by intraperitoneal LPS in mice, which causes systemic inflammation rather than amniotic fluid inflammation. Data in the non-human primate demonstrate that infectious agents confined to the choriodecidua induce preterm labor at a much lower frequency compared to infection in the amniotic fluid (81–83). Our data demonstrate that macromolecules such as cytokines (IL1ra) do not efficiently cross the amniotic epithelial barrier but readily cross the placenta from the mother to the fetus (84). Thus, the evaluation of compartment-specific mechanisms of inflammation is important. Indeed, differentially expressed genes in spontaneous preterm birth compared to gestational controls had different transcriptomic profiles in different tissues (85). These results are consistent with different tissue-specific roles in the intrauterine space. In a prospective study with detailed phenotyping of preterm and term labor, the largest difference in gene expression groups occurred at the maternal fetal-interface in the decidua, chorion, and amnion (86).

During intrauterine infections, ascending organisms are thought to spread diffusely through the choriodecidual or the chorioamnion plane and then invade the amniotic cavity. However, a study using molecular microbiologic techniques in human placentae demonstrated that the initial event is a localized choriodecidual infection, which then invades the amniotic cavity, infecting the amniotic fluid and fetus prior to causing diffuse choriodecidual inflammation (87).

## Animal Models

Several animal models have been reported for IUI (see Table 1 with representative references for each model). Each model has some advantages but also important limitations. Mice are commonly used in reproductive research, have the advantage of being genetically modifiable to enable mechanistic studies, and are also relatively inexpensive. Other species include rats, rabbits, guinea pigs, sheep, non-human primates and others. A major advantage of non-human primates, mice, rats, and guinea pigs is that all of them have hemochorial placenta, while sheep have epithelio-chorial placenta. In all of these animal models, IUI can be induced by injecting different pathogen-associated molecular pattern molecules (PAMPs), damage-associated molecular patterns (DAMPs), or live microorganisms (107). The characteristic features of acute chorioamnionitis are diffuse infiltration of neutrophils into the chorion and amnion membranes, and increased inflammatory cytokines and chemokines (108).

**Table 1:** Animal models for IUI.

**Animal model**	**Route of injection**	**Pathogen/agonist**	**References**
Mouse	i.p.	TLR agonists: LPS, Poly I:C, Pam3Cys, Pam2Cys, LTA; Bacteria: *E. coli*, heat-killed *E. coli*, GBS; Viruses: MHV-68, LMCV; Parasites: Toxoplasma gondii	(65, 67, 88–92)
	i.v.	Listeria monocytogenes; Salmonella Typhimurium; Fusobacterium nucleatum; Chalmydophiula abortus	(49, 67, 93–95)
	i.n.	Influenza	(67)
	i.u. (surgery)	LPS	(96)
	i.u. (ultrasound-guided)	LPS	(88)
	i.a. (ultrasound-guided)	LPS	(97)
	i.vag.	*E. coli*, LPS	(88, 98)
NHP	i.a.	LPS, Ureaplasma species, GBS	(82, 84, 99)
	Choriodecidual space	GBS	(82)
	Subcutaneous, i.a., i.v.	ZIKV	(100–102)
Sheep	i.a.	LPS, Ureaplasma species	(103–105)
	i.v.	LPS	(104)
	Subchorionic	LPS	(106)

*i.p., intraperitoneal; i.v., intravenous; i.n., intranasal; i.u., intrauterine; i.a., intraamniotic; i.vag., intravaginal*.

The route of injection is important since TLR activation in different compartments elicits different responses. Systemic (intravenous, intraperitoneal, or subcutaneous) injection of agents will cause a systemic inflammatory response. In mice, systemic inflammation can cause progesterone withdrawal due to the regression of the corpus luteum, triggering preterm birth (109). Localized intrauterine inflammation can be induced in mice by injecting agents into the uterine horn between the gestational sacs (88). Most commonly, this has been done by mini-laparotomy to expose the uterine horns (96); however, such procedure could induce adverse pregnancy outcomes due to a rapid localized inflammatory response that surrounds the incision area. In the last few years, new less-invasive techniques have been developed, including ultrasound-guided intraamniotic and intrauterine injection of TLR, thus minimizing the effects of surgery (88). Similarly, ultrasound-guided intraamniotic injection has been used in a variety of species. Other non-invasive methods including intravaginal injection/inoculation have also been used (88, 98).

In non-human primates, both intraamniotic or choriodecidual injection/infusion of agents has been used. In some studies, the animals are placed in a nylon jacket and tether with the catheters/electrodes tracked through the tether system (with 360° mobility), requiring prior adaption of the animal and considerable animal handling skills (31). Although sheep have been traditionally used for study of fetal physiology and fetal inflammation, they are not suited for preterm labor studies since they lack the decidua and the placental architecture is considerably different from humans (110). Thus, while interpreting and comparing studies, the species used, route of injection, dosage of agents, and other experimental details need to be carefully considered to correctly interpret experimental data.

## Immune and Non-Immune Cells at the Feto-maternal Interface

Several distinct immune cells populate the normal maternal-fetal interface with distinct homeostatic roles during normal pregnancy (111). Immune cells in the choriodecidua have distinct immunological profiles and functions compared to their counterparts in blood (112). However, the repertoire of immune cells changes during IUI and parturition (113) and will be reviewed below (Table 2).

**Table 2:** Immune and non-immune cells at the feto-maternal interface during IUI.

**Cell type**	**Species**	**Phenotype/mechanism**	**References**
Neutrophils	Human	In AF of women at term with acute chorioamnionitis neutrophils are the most abundant populations and produced predominantly TNFα and MIP1-β/CCLA4	(114)
	Human	IL-8 and CXCL6-dependent migration of both maternal and fetal neutrophil into the chorioamniotic membranes	(20, 115)
	Non-human primate	40% of choriodecidua neutrophils express IDO1 in IA-IL-1β injected animals	(116)
	Non-human primate	IA LPS induced expression of the prosurvival factor BCL2A1/BFL1 in choriodecidua neutrophils is IL-1 dependent	(84)
	Sheep	Repeated LPS-exposure decreased numbers of fetal lung neutrophils and iNOS expression	(117)
	Mice	Intrauterine infection with *U. parvum* causes an extensive neutrophilc infiltration undergoing necrosis and responsible of fetal infection in BALB/c mice and not in C57BL/6 mice	(118)
Monocytes/macrophages	Human	Accumulation of CD14^+^CD163^+^DC-SIGN^+^ macrophages in fetal membranes during acute chorioamnionitis	(119)
	Human	Higher frequency of CD16^+^CD206^+^Arg-1^+^ M2 macrophages compared to M1 macrophages in acute chorioamnionitis	(120)
	Human	Placental macrophages infected *ex vivo* with GBS release extracellular traps containing MMPs and capable of killing GBS cells	(121)
	Non-human primate	CD68^+^ macrophages accumulated in uterine tissues upon IA *U. parvum*-exposure	(99)
	Non-human primate	Number of CD14^+^HLA-DR^+^ monocytes/macrophages increased during chorioamnionits caused by IA injection of LPS	(84)
	Sheep	7 days after IA LPS exposure blood and lung monocytes secreted high levels of IL-6 and H_2_O_2_ upon *in vitro* LPS challenge compared with monocytes from control lambs	(117)
	Mice	Notch signaling induce the polarization of decidual macrophages toward CD11c^+^M1^+^ and CD11c^+^M1^+^/CD206^+^M2^+^ double positive macrophages in intrauterine LPS-treated mice and Peptoglycan^+^poly(I:C)-induced PTL.	(122, 123)
ILCs	Human	Increased proportion of total CD15^−^CD14^−^CD3^−^CD19^−^CD56^−^CD11b^−^CD127^+^ ILCs in the human decidua parietalis associated with spontaneous preterm labor	(124)
Tregs	Human	Increased numbers of human cord blood FOXP3^+^ROR+IL-17^+^ Tregs in live birth neonates with acute chorioamnionitis	(125, 126)
	Human	Cord blood Ki67^+^Tregs showed lower levels of suppression compared to term or preterm without or with mild chorioamnionitis	(127)
	Non-human primate	Higher frequency of fetal spleen FOXP3^+^IL-17^+^IL-22^+^ Tregs in IA LPS model of chorioamnionits compared to IA saline controls	(128)
	Non-human primate	Frequency of CD127-CD8-CD25^+^FOXP3^+^ choriodecidua Tregs did not change upon IA IL-1β-injection	(84)
	Sheep	In IA IL-1α, *U. parvum*- or LPS-exposed lambs the numbers of FOXP3^+^ Tregs decreased in peripheral blood and fetal lymphoid tissues, including spleen, lymph nodes, gut, and thymus	(104, 129–132)
	Mice	The adoptive transfer of Tregs in IP LPS-injected mice at gd 17 significantly suppressed the LPS-induced inflammatory response in the fetal brain by decreasing the expression of Foxp3, IL-6, and TLR-4	(133)
NK	Human	Increased frequency of CD16^+^NK cells in decidua basalis in women that underwent PTL with acute chorioamnionits resulted from the recruitment of circulating NK cells	(134)
	Human	Decreased frequency of choriodecidua CD3-CD14-CD56^+^ NK cells in women who underwent preterm with chorioamnionits compared to those ones who went preterm without chorioamnionits	(84)
	Non-human primate	In cord blood and fetal spleen IA U. parvum exposure did not alter the frquency of CD3–CD8–NKG2A^+^ NK cells	(99)
	Non-human primate	IA IL-1β or LPS injection at ~80% of gestation did not alter the numbers of CD56^+^ dNK cells	(84)
	Mice	IP LPS injection at gd 15 caused an up-regulation of activated CD69^+^CD49b^+^ NK cell proportion at feto-maternal interface	(135)
DCs	Human	DEC-205^+^CD86^+^ DCs from women who underwent preterm birth with acute chorioamnionitis induced proliferation of NK cells	(134)
	Human	Blood HLA-DR^+^CD11c^+^ IL-12/23p40^+^ DCs frequency does not change upon *in vitro* stimulation with LPS in very preterm neonates with histological chorioamnionitis compared to term neonates or adult have been reported	(136)
	Non-human primate	No changes in frequency as well as activation status of both pDCs and mDCs in peripheral blood and lymphoid tissues of *U. parvum* exposed fetus	(99)
	Mice	In IP LPS-injected mice at gd 17-18, the frequency of MHCII^low^CD11c^hi^ DCs was significantly elevated in livers but not altered in lungs or spleens in postnatal day 2 pups	(137)
B cells	Human	Slight increase of decidual plasmablasts and B1 B cells in women who underwent labor at term or preterm with chronic chorioamnionitis compared to those with acute chorioamnionitis or without it	(138)
	Non-human primate	CD19^+^CD20^+^ B cells numbers are ~1.5-fold increase in choriodecidua of IA LPS-injected Rhesus macaque compared to IA saline-injected controls	(84)
	Mice	B cells conferred resistance to inflammation-driven PTL independently of IL-10 in the third trimester in mice	(139)
	Mice	IV injection of IL-10 producing B cells at gd8 to pregnant mice lacking of mature B cells before IP LPS challenge (at gd10) restored tolerance	(140)
iNKT	Human	Increased frequency of CD3^+^CD56^+^Va24^+^ iNKT cells in women that underwent pre-term labor without acute chorioamnionitis compared to those who went pre-term labor with chorioamnionitis	(134)
	Human	Accumulation of CD3^+^CD56^+^CD69^+^ NKT cells in the decidua basalis of women that underwent preterm labor without intra-amniotic infection compared to those ones that deliver preterm without labor	(141)
	Mice	Depletion of iNKT In IP LPS-injected mice at 15dpc decreased expression of costimulatory molecules CD40, CD80, and CD86 in decidual DCs, and suppressed the expansion and activation of decidual NK cells	(142)
	Mice	Adoptive transfer through IV injection of decidual iNKT cells into LPS-stimulated Ja18^−/−^ mice significantly induced PTB by recruiting adopted iNKT cells into the decidua	(143)
Trophoblasts	Human	Increased villous trophoblast Fas-mediated apoptosis	(144)
	Non-human primate	Pancytokeratin^+^ trophoblast cells did not produce CXCL8/IL-8 upon IA-LPS injection at ~80% of gestation	(116)
	Mice	Intraperitoneal Poly[I:C] injection on 16.5 dpc caused activation of trophoblast cells that produce KC, GM-CSF, IL12p40, MIP-1α, MIP1-β, and this response was mediated through TLR3 expression and function	(145)

### Neutrophils

Neutrophilic infiltration of choriodecidua and amnion is a characteristic feature during IUI and forms the basis for the pathological definition of chorioamnionitis (146). Neutrophils infiltrating the choriodecidua are largely of maternal origin (147, 148), while neutrophils in the amniotic fluid are largely of fetal origin (149), although a recent study reported mixed fetal/maternal origin of neutrophils in the amniotic fluid (150). During normal pregnancy, there are small numbers of neutrophils, but the numbers increase dramatically during IUI presumably due to secretion of neutrophilic chemotactic factors such as IL-8 by the amnion (84, 108). CD15^+^ neutrophils represent the most abundant leukocyte population in AF from women with intraamniotic infection/inflammation (151). During IUI, the choriodecidual neutrophils predominantly produce TNFα, IL-8, and MIP-1β/CCL4 (114, 116). Although blood neutrophils have a short life-span, neutrophils in the decidua have greatly increased survival, mediated in part by up-regulating anti-apoptotic mediators including Bcl-2 family members (e.g., Bcl2A1, called Bfl-1 in humans) (84, 152). Neutrophils can amplify the inflammatory response and neutralize microorganisms by releasing neutrophil extracellular traps (NETs), producing antimicrobial enzymes such as defensins, neutrophil elastase, and myeloperoxidase (MPO), and releasing reactive oxygen species (ROS) (153–155). Neutrophil-derived pro-inflammatory mediators can trigger preterm labor and matrix metalloproteinases (MMPs) can weaken the collagen scaffolding, resulting in preterm rupture of membranes (156–158). However, studies in mice with systemic neutrophil depletion did not protect against inflammation-induced preterm birth (159, 160). Although the reasons for the apparent contradiction are not clear, neutrophil depletion in mice causes a systemic up-regulation of G-CSF, IL-17, and IL-23, leading to hyper-cellular bone marrow as a homeostatic “neutrostat” mechanism to restore the blood neutrophil pool (161, 162). Thus, strategies other than neutrophil depletion are needed to understand the role of neutrophils in triggering preterm labor.

Although neutrophils are classically considered to be pro-inflammatory, regulatory neutrophils have been increasingly recognized in different tissues (163). In the decidua, neutrophils expressing anti-inflammatory indoleamine 2,3-dioxigenase 1 (IDO1) (116), and pro-angiogenic vascular endothelial growth factor (VEGF) are previously postulated to play a role in cyclic vascular proliferation in the endometrium (164, 165), and in 1st and 2nd trimester human decidua basalis (166, 167). Based on peak neutrophil influx after the onset of normal labor, post-partum uterus/decidua matrix remodeling and wound healing function has been attributed to decidual neutrophils (113, 168–170). Thus, there appears to be neutrophil heterogeneity and a role for neutrophils in tissue homeostasis at the maternal-fetal interface similar to that for other tissues (163, 171).

### Monocytes/Macrophages

Monocytes/macrophages represent 20–30% of the leukocyte population at the maternal-fetal interface. These include macrophages of maternal origin in the decidua, and the Hofbauer cells of fetal origin in the placental villi (172, 173). Monocytes/macrophages in the amniotic fluid, especially in cases of IUI, can be of mixed maternal/fetal origin (174). Placental macrophages are essential for normal pregnancy as genetic depletion of CD11b+ macrophages prevents embryo implantation via regulation of corpus luteum development in mice, and the csf1-deficient osteopetrosis mice have severe infertility (175). Decidual macrophages play a key role in angiogenesis, tissue remodeling, immune surveillance, host-defense, antigen-presentation, and immune tolerance. For example, decidual macrophages inhibit NK cell-mediated lysis of human cytotrophoblasts by TGF-β1 secretion (176), and by phagocytosis of apoptotic cells (177). Macrophages also play a key role in decidualization, angiogenesis, uterine contraction, and tissue remodeling (168, 173, 178).

Since decidual macrophages perform diverse functions, the polarization varies from being largely pro-inflammatory (M1-like) during the peri-implantation period, anti-inflammatory (M2-like phenotype) during mid to late pregnancy, and pro-inflammatory (M1-like phenotype) during parturition (179). However, decidual macrophages cannot be strictly phenotyped as either M1 or M2. Rather, Houser et al. described two unique decidual macrophage populations based on their CD11c expression (180). Recently, these observations were further clarified with the description of three decidual macrophage subsets, CCR2^−^CD11c^low^ in the decidua parietalis, and CCR2^−^CD11c^hi^ and CCR2^+^CD11c^hi^ in proximity of extra villous trophoblasts during the first trimester of human pregnancy. Although the three different subsets exhibit phagocytic capacity, CCR2^−^CD11c^low^ macrophages showed an M2-like anti-inflammatory phenotype while CCR2^+^CD11c^hi^ an M1-like pro-inflammatory phenotype, suggesting that those different macrophage subsets contribute to maintaining an inflammatory balance at the maternal-fetal interface (181).

During IUI, decidual macrophage numbers increase (84), with a predominance of M2-like phenotype in humans (120). Similar to neutrophil NETs, macrophage extracellular traps were recently reported in the decidua in response to group B Streptococcus infection (121). These traps containing MMPs effectively kill invading microorganisms, thereby protecting the host. Although not a topic of this review, disruption of appropriate macrophage polarization is also associated with other abnormal pregnancies, including spontaneous abortions and preeclampsia (179).

### T Cells

T cells play an important role in the setting of IUI and are involved in the mechanisms responsible of induction of labor (182–184). *In vivo* T cell activation by intraperitoneal injection of αCD3ε antibody in late gestation causes pathological inflammation and initiates innate and adaptive immune responses which, in turn, lead to preterm labor and birth (185). Our group recently reported an increase of CD3^+^ T cells in uterine tissues after intraamniotic *Ureaplasma* exposure in Rhesus macaques (99). T cells represent ~20–30% of CD45^+^ cells at the maternal-fetal interface in a Rhesus model of LPS-induced IUI as well as in human preterm pregnancy (84). In humans, more CD45RO memory T cells accumulated in the choriodecidua at term (regardless of the presence of labor) than in preterm pregnancy without labor in a CXCL10/CCL5-dependent manner (184, 186). These cells express high levels of MMP-9, IL-1β, and TNFα (184), cytokines involved in the onset of labor as well as mechanisms of membrane rupture (187).

During IUI, maternal CD8 T cells are increased in both the placenta and peripheral circulation and they express CCR3, perforin, and granzyme B (188–190). CD8^+^ T cells have the ability to kill fetal cells and therefore should be excluded from the maternal-fetal interface. Indeed, Nancy et al. showed that impaired effector T cell accumulation in the decidua is partly mediated by epigenetic silencing of key T cell-attracting inflammatory chemokines (11) during normal pregnancy. However, after *L. monocytogenes* prenatal infection, maternal CD8^+^ T cells with fetal specificity selectively upregulate the expression of CXCR3, CXCL9 receptor, and are recruited to the decidua by CXCL9-secreting neutrophils (93). CXCR3 blockade before or shortly after *L. monocytogenes* infection correlated with fewer CD8^+^ T cells in the decidua and no fetal death (93).

### Maternal-Fetal Tolerance and Tregs

A number of protective mechanisms have been identified to explain the immunologic paradox of maternal tolerance of the semi-allogenic fetus (191). These include: (1) fetal cells in contact with maternal cells expressing non-classical MHC antigens; (2) maternal NK cells being less cytotoxic and recognizing the fetal trophoblasts with non-classical MHC (192–194); (3) the role of decidua stromal in local immune modulation and maintenance of immune tolerance (11, 195); (4) a skew toward anti-inflammatory macrophage phenotype; (5) disappearance of lymphatics from the endometrium upon decidualization (196); and (6) chemokine silencing to decrease trafficking of cytotoxic T-cells in the decidua (11).

Regulatory T-cells (Tregs) play a pivotal role in maternal-fetal tolerance (197). Immature dendritic cells induce Treg formation by secreting IDO and TGFβ (198, 199). Decidual NK cells can also facilitate the generation of Tregs (199, 200). Regulatory B cells (Bregs) are the B cells producing IL-10 under the influence of gonadotropins. Bregs are increased in early human pregnancy and suppress TNFα production by T cells (201, 202). Maternal Tregs, stimulated by chorionic gonadotrophins, accumulate at the maternal-fetal interface during early gestation (203, 204), peak in the second trimester (205), and mediate tolerance to paternal antigens, facilitating embryo implantation and preventing embryo resorption (206–209).

Immune tolerance of the fetus during pregnancy places the mother at a higher risk for impaired host defense. Indeed, Treg expansion in mouse models increased susceptibility to Listeria and Salmonella infections during pregnancy (210). Apart from fetal immune tolerance, Tregs also suppress activity of other decidual immune cells. Indeed, Treg secretion of TGFβ and IL10 downregulate decidual NK cell activation, and this suppression is protective against pregnancy complications such as pre-eclampsia and recurrent abortions (211, 212). However, the numbers of CD3^+^CD8^−^FOXP3^+^ Tregs at the maternal-fetal interface did not change upon intraamniotic LPS exposure in Rhesus macaques (84). Treg phenotype in the fetus during chorioamnionitis is discussed elsewhere in this journal.

### NK Cells

In the first trimester, decidual natural killer cells (dNK) cells comprise ~70% of immune cells at the choriodecidua, representing the largest population of leukocytes at the maternal-fetal interface. Both immunological phenotypes and function of decidua NK cells differ from their blood counterparts. Compared with circulating CD56^bright^CD16^+^ NK cells, CD56^bright^CD16^−^dNK cells present a lower cytotoxicity and higher levels of killer immunoglobulin-like receptors (KIRs) and natural killer group 2 (NKG2) receptors (192, 213). dNK cells express exclusively CD151, CD9, and tetrasparan-5 compared to peripheral NK cells in the first trimester of gestation (214). After implantation, CXCR4^+^ NK progenitor cells migrate into the uterus via a CCL3/CXCL12 gradient (215), and proliferate in response to IL-15 (216). The number of the uterine NK cells decreases significantly at gd 6 in mice (217), peaks at gd10 (218), and decreases dramatically at d12, when the majority of NK cells have become senescent (219). Uterine NK cell-derived IL-8, CXCL10, IFNγ, and vascular growth factors are critically needed for decidual spiral artery remodeling and successful pregnancy outcomes 149, (220–222). Decidual NK cells play an important role in recognizing paternal MHC on trophoblast cells via killer-cell immunoglobin-like receptor interactions, mediating tolerance (192–194). Furthermore, a cross-talk between dNK and dCD14^+^IDO^+^ cells could promote the generation of Tregs, thereby facilitating fetal immune tolerance (199, 200).

Despite important roles performed by NK cells during normal pregnancy, the role of these cells is not clear in the pathogenesis of chorioamnionitis. In mice, intraperitoneal LPS injection increased the frequency of activated NK cells CD69^+^CD49b^+^ at the maternal-fetal interface (135), while we did not demonstrate changes in NK cell frequency in the choriodecidua of Rhesus macaques given intraamniotic LPS (84). In humans, both decidua basalis and parietalis showed an increased frequency of CD56^+^NK cells in women that underwent pre-term labor with acute chorioamnionitis compared to those without chorioamnionitis (134). Although NK cells perform homeostatic roles, under pathological conditions such as fetal alloimmune thrombocytopenia, dNK cells can become activated with prolonged survival, elevated NKp46 and CD107 expression, and perforin release, and can induce trophoblast apoptosis and placental pathology, leading to miscarriage (223).

### Dendritic Cells (DCs)

DCs are instrumental for decidualization and angiogenesis in mice because their depletion prevents blastocyst implantation and formation of the decidua (224, 225). Large amounts of DEC-205^+^ DCs accumulated in both the decidua basalis and parietalis obtained from women who underwent preterm birth regardless of the presence of acute chorioamnionitis (134). Peripheral blood myeloid and plasmacytoid DCs numbers fall in the second trimester but subsequently increase in the third trimester (226, 227) and become more activated (228, 229). These DC populations show lower levels of costimulatory molecules CD40, CD80, and CD86 during pregnancy complications compared to healthy pregnancy (230).

Little is known about the role of DCs during chorioamnionitis. A number of studies in experimental models and humans demonstrate minimal changes in circulating DCs during chorioamnionitis (99, 136). Interestingly, DEC-205^+^ DCs obtained from women who underwent preterm birth without acute chorioamnionitis had a higher expression of the costimulatory molecule CD86 and produced more IL-12. These DCs preferentially enhanced the proliferation of iNKT cells *in vitro* (134). On the contrary, DEC-205^+^ DCs from women who underwent preterm birth with acute chorioamnionitis induced proliferation of NK cells (134).

### B Cells

Although B-cell deficient mice have normal pregnancies, IL10 producing B cells protected fetuses from intrauterine demise when exposed to LPS (140). In human term and preterm pregnancy, total CD19^+^ B cells represent a small fraction (<5%) of decidual leukocytes in both the decidua parietalis and basalis (138, 231). Furthermore, B cell frequency did not significantly change during the course of pregnancy in decidua basalis even during systemic inflammation (231). However, the frequency of CD19^+^ B cells were higher in the decidua parietalis of women who underwent preterm birth regardless of labor (138, 139). These results are consistent with our results of a ~1.5-fold increase in CD19^+^CD20^+^ B cells numbers in the decidua parietalis in Rhesus macaque exposed to intraamniotic LPS compared to IA saline controls (84). Functionally, B cells were reported to be protective for preterm birth in mice via IL-33 and progesterone-induced blocking factor, decreasing LPS induced neutrophil recruitment (139), although this protective action has been questioned in a recent study (138).

Decidual B cells can secrete both pro-inflammatory IL-12 and IL-6, as well as immunosuppressive IL-35 (138) suggesting multiple context-dependent roles for B cells during intrauterine inflammation. Furthermore, CD19^+^CD24^hi^CD27^+^ B cells can produce IL-10 under the influence of gonadotropins, and these regulatory B cells (Bregs) are increased in early human pregnancy and suppress TNFα production by T cells (201, 202). Additionally, adoptive transfer of IL-10 producing B cells increased the number of uterine Tregs and protected against LPS-induced adverse pregnancy effects by decreasing the production of IL-17A and IL-6 by naïve CD4^+^CD25^−^ T cells (140).

### iNKT Cells

Invariant natural killer T cells (iNKT) specifically recognize glycolipid antigens presented by MHC class I-related protein CD1d and produce large amounts of both Th1 and Th2 cytokines upon stimulation (232). iNKT cells are present in both human and mouse decidua (233–236), and their specific ligand CD1d is expressed in human villous and invasive extra villous trophoblast (EVT) (235). Indeed, injection of the CD1d ligand α-galactosylceramide (αGalCer) resulted in iNKT cell-mediated pregnancy loss that was dependent on gestational age, strain, and route of administration (141, 233, 235). Furthermore, placenta from women with non-infectious preterm delivery demonstrated an increase in activated iNKT cells (134, 141, 237). Although the mechanism is not clear, one possibility is that iNKT mediates the activation of decidual DC, and increases the expression of co-stimulatory molecules CD40, CD80, and CD86 (142). Moreover, Jα18^−/−^ mice lack iNKT cells and are resistant to LPS-induced preterm birth. Further support of the role of iNKT in inflammation-mediated preterm delivery in mice is that adoptive transfer of decidual iNKT cells into LPS-stimulated Jα18^−/−^ mice significantly induced preterm birth by recruiting adopted iNKT cells into the decidua (143). Overall, the results suggest that activation of iNKT cells in the decidua may play a role in triggering preterm birth of non-infectious etiology.

### ILCs

Innate lymphoid cells (ILCs) are a group of immune cells belonging to the lymphoid lineage, although they do not express antigen-specific receptors. These cells are found at the mucosal surface and are extremely important in innate immune responses to infectious microorganisms and in the regulation of homeostasis and inflammation (238). To date, three distinct subsets of ILCs have been identified based on their different phenotype and functions: ILC1s (dependent on the expression of T-bet); ILC2s (dependent on the expression of GATA3 and RORα); and ILC3s (dependent on the expression of RORγ) (239). ILC2 cells are the most common ILCs expressed in preterm and term decidua (124). However, their role in the context of acute chorioamnionitis is not well-defined.

In preterm birth patients, an increase in both ILC2s and ILC3s were observed in the decidua basalis and decidua parietalis, respectively (124). Interestingly, ILC3s in the decidua from women with spontaneous preterm labor were activated since they expressed higher levels of IL-13 and IFNγ, cytokines normally produced by ILC2s and ILC1s, respectively (124, 240). Further studies are needed to elucidate the biology ILCs during normal and complicated pregnancies.

### Trophoblast Cells

The trophoblast cells in villous placenta include the cytotrophoblast and syncytiotrophoblast. In extraplacental fetal membranes, the trophoblast cells are called EVTs. Fetal trophoblasts are a component of innate immune system and play an important role in orchestrating the maternal innate immune response to infection at the maternal-fetal interface (241, 242).

Fetal trophoblasts employ several mechanisms to suppress maternal immune cells: (1) HLA-G protects EVTs from NK-cell mediated cytotoxicity (243); (2) HLA-G^+^ EVTs express anti-inflammatory mediators including IDO (244), programmed death ligand PDL-1 (245); IL-10 and TGFβ (246, 247); (3) EVTs enhance the expansion of maternal Tim3^+^PD-1^+^CD8^+^ T cells in the decidua which recognize PDL-1^+^ EVTs and downregulate cytotoxicity (248); (4) HLA-G^+^ EVTs induces tolerogenic DCs by disruption of the MHCII presentation pathway, inducing differentiation of anergic and immunosuppressive CD4^+^ and CD8^+^ effector T cells (249); (5) Cocultures of HLA-G^+^ EVTs with sample matched decidual CD4^+^ T cells results in increased number of Tregs (250). How these mechanisms are altered in the context of IUI is not known.

Studies in experimental models clearly demonstrate that the trophoblasts are capable of response to an inflammatory challenge and modulate the immune response. In mice, intraperitoneal poly[I:C] injection induces stimulation of TLR3, and activates trophoblast cells through the production of KC, GM-CSF, IL-12p40, MIP-1α, and MIP-1β (145). *In vitro*, first trimester trophoblast cells can promote monocyte migration through the production of GRO-α, MCP-1/CCL2, and CXCL8/IL-8 in response to LPS (251, 252). Interestingly, the trophoblast-activation response to LPS was dose dependent. Compared to high doses of LPS, low doses of LPS result in the lesser production of chemokines and cytokines, and therefore less trophoblast activation (251, 252). Trophoblast cells can also release high amounts of IL-8 that triggers NET formation in preeclampsia (253). On the other hand, trophoblast cells can inhibit neutrophil activation, survival, NET formation, and ROS synthesis via vasointestinal peptide and other glucose metabolism pathways (254, 255).

### Amnion

As the amnion is in contact with AF, it is strategically located to transduce inflammatory signals in the AF to mount the immune response (84). In a Rhesus macaque model of IUI, the amnion upregulated phospho-IRAK1-expressed neutrophil chemoattractants CXCL8 and CSF3 in an IL-1-dependent manner (84). Moreover, amniotic cells express a set of TLRs suggesting that the amnion plays an important role as sentinel cells that recognize a wide variety of pathogen derived molecular patterns (26, 256). When stimulated or exposed to inflammatory signals, the amnion can also secrete pro-inflammatory mediators including prostaglandins and cytokines/chemokines (256, 257). Thus, it appears that the amnion may be a sensor and regulator of the inflammatory response to infectious/inflammatory stimuli and play a role in triggering preterm labor.

### Decidua Stroma Cells

The decidual stromal cells express immune receptors that respond to ascending infections during pregnancy (258). Stromal cells are in contact with multiple different immune cells and modify the decidual immune response. Their role in during IUI needs to be better defined. In *in vitro* studies, decidual stromal cells exerted a powerful inhibitory effect on NK cell proliferation and DC differentiation (259). Decidual stromal cells can also interact with macrophages in regulating the immune response against pathogens through the release of PGE2 and IL-8 (260, 261). Moreover, decidual stromal cells also regulate the interplay between pro-inflammatory cytokines and the reproductive endocrine system that may modulate inflammation-mediated preterm birth (262). Thus, decidual mesenchymal cells, like mesenchymal cells in other niches, can both downregulate or upregulate the activity of decidual immune cells in a context-dependent manner.

## Pathogenesis of Chorioamnionits and Potential Therapeutic Strategies

**Figure 3:**
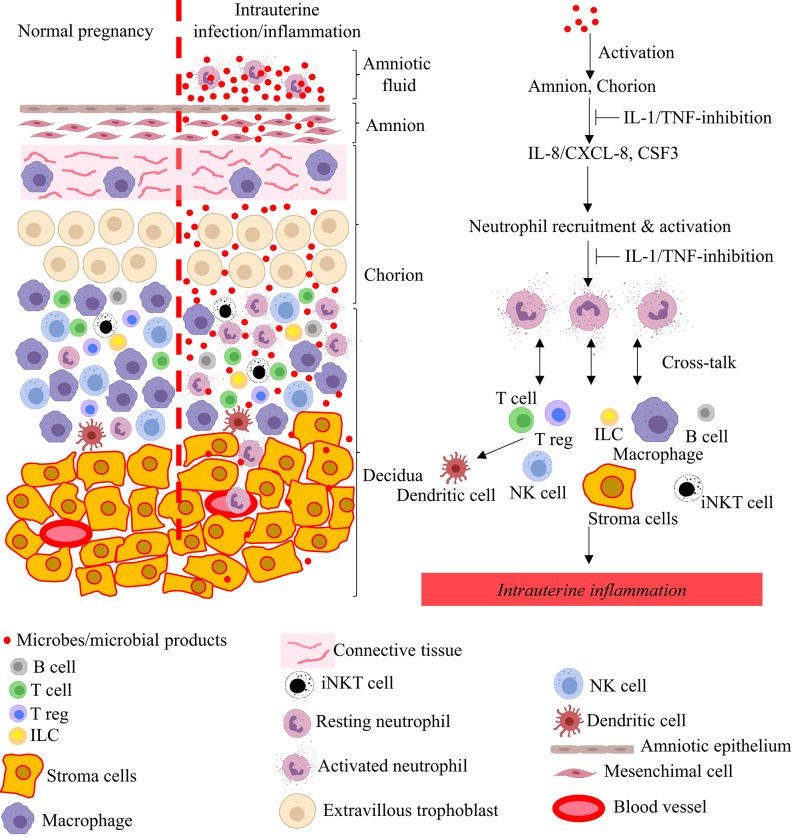
Model for pathogenesis of intrauterine infection/inflammation. Representative cells in the different tissue layers of fetal membrane are shown. The left panel in figure depicts normal pregnancy and the right panel shows changes during IUI. Inflammatory products and microbial products (red dots) in the amniotic fluid and choriodecidua activate the amnion and chorion, resulting in the release of neutrophil chemoattractant (CXCL-8/IL-8 and CSF3) in a IL-1 and TNF-dependent manner. Neutrophils accumulate at choriodecidua junction, get activated, and greatly amplify the inflammation at the maternal-fetal interface with cross-talk with other immune and resident cells.

As mentioned above, the pathological hallmark of chorioamnionitis is neutrophil infiltration in the fetal membranes, and is often associated with neutrophils in the amniotic fluid. Although the mechanisms of neutrophil recruitment at the maternal-fetal interface is not entirely clear, the amnion may play an important role by secreting chemoattractants (84). Neutrophils that accumulate at the maternal-fetal interface are activated with increased survival mediated by the anti-apoptotis factors belonging to the Bcl family (84). The interplay between neutrophils, macrophages, Tregs, CD8 T cells, and the decidual stromal cells regulate the intensity of inflammation, and secretion of cytokines, chemokines, and prostaglandins (Figure 3). The net result is a feed-forward loop of inflammation that can result in preterm labor and birth. Viral infections or activation of the type-I interferon signaling can further potentiate inflammation (61, 65, 67, 145). If the inflammatory stimulus subsides, resolution of the inflammatory process can also occur.

Antibiotics, the mainstay of the current treatment, are largely ineffective in preventing IUI-associated morbidities (263, 264), partly because residual inflammation from the infection can cause fetal and maternal injury (100, 128, 265, 266). Therefore, the development of alternative therapeutic approaches is essential (267).

The nuclear factor-κB (NF-κB) proteins are prototypic molecules involved in inflammation and immune signaling. Upon activation by a variety of stimuli including LPS, the normally inactive NF-κB proteins retained in the cytoplasm by IκB, are activated and translocate to the nucleus, where they increase the transcription of target genes (268). Since NF-kB plays a pivotal role in cellular inflammatory response, several NF-kB inhibitors have been tested to block IUI. N-acetyl-cysteine (NAC) inhibits inflammation in human fetal membranes *in vitro* (269) and *in vivo* (270–272). In a clinical trial, NAC administered to women between 16 and 18 weeks' gestation with previous preterm labor and bacterial vaginosis reduced the recurrence of preterm birth (273). High concentrations of sulfasalazine, another suppressor of NF-kB activity, reduced inflammation but also induced apoptosis in the chorion in an *ex-vivo* model of fetal membrane inflammation (274). Cytokine-suppressive anti-inflammatory drugs (CSAIDs) specifically target inflammatory signaling pathway such as NF-kB, and are therefore candidates for the treatment of IUI. TPCA-1 and parthenolide are selective inhibitors of the kinase complex that regulates the NF-κB cascade, such as IKKβ. Both TPCA-1 and parthenolide inhibited human choriodecidual IL-6 and TNFα production and inflammatory gene expression *in vitro* (275). Similarly, in an ovine model of IA LPS-induced chorioamnionitis, TPCA-1 and 5z-7-oxozeaenol abrogated the stimulatory effects of LPS on prostaglandin production in the AF. However, fetal lung inflammation was not affected by the treatment of those two compounds, suggesting that the beneficial effects on the fetus were minimal (276). Non-steroidal anti-inflammatory drugs (NSAIDS) are another class of compounds used in the treatment of inflammation with some success in Rhesus macaque models (277, 278), but they are not without risks for the fetus (279).

TNFα is a major pro-inflammatory cytokine whose levels are increased during IUI. TNFα blockade decreased adverse pregnancy outcomes in rodents (280, 281). A small human study demonstrated that TNFα-blockade can improve outcomes in women with recurrent spontaneous abortions (282). However, a concern with using the clinically approved anti-TNFα antibodies is that the drug freely crosses placenta and is detectable in the infant after birth since the half-life is several weeks, and prolonged inhibition of TNFα can result in immune suppression. Another important cytokine implicated in IUI is IL-1β (277, 283–285). In a variety of animal models, inhibitors of IL1 signaling such as Anakinra (recombinant IL-1 receptor antagonist) and peptide inhibitors substantially reduced intrauterine neutrophil infiltration and inflammation (84) and fetal inflammation (3, 128, 286–290). However, the efficacy of IL-1 inhibitors in preventing preterm birth has been questioned (284, 291). An attractive feature of the widely used clinical drug Anakinra (recombinant IL-1 receptor antagonist) is that it has a short half-life, thus decreasing the concern for prolonged immunosuppression of the fetus. Anakinra is widely used as an anti-inflammatory agent for Rheumatoid arthritis and other inflammatory diseases (292), and classified as a class B drug during pregnancy by the US FDA (No harm to the fetus in animal studies but lack of well-controlled studies in humans). Anecdotal use during pregnancy has been reported the drug to be safe but well-controlled trials are lacking (293).

## Conclusions

Although the link between chorioamnionitis and the risk for maternal and fetal health has long been recognized, important questions remain about the immunobiology of IUI. Multiple lines of evidence from animal experiments and in humans have convincingly demonstrated that different microorganisms can cause IUI through various routes of invasion. Moreover, the emerging concept of priming viral infections and polymicrobial infection need to be further investigated.

Among different immune cells, neutrophils infiltrating the chorioamnion decidua tissue play a major role in the pathogenesis of IUI. The mechanisms regulating neutrophil recruitment to fetal membranes and their role in promotion of IUI currently represent an area of active investigation.

Antibiotic therapy for IUI has been disappointing so far, likely because of the residual intrauterine inflammation. There is a clear need to develop new intervention strategies aimed at reduction of the morbidity and mortality associated with IUI.

Lastly, more efforts are needed to build interdisciplinary teams spanning reproductive biology, infectious diseases, pharmacology and immunology, maternal and fetal health that would allow a broad approach in the understanding of the pathogenesis of chorioamnionitis and to develop new therapeutics to prevent/cure IUI.

## Author Contributions

MC, PP, and SK contributed to the scope and setup of and wrote the review. All authors approved the final version.

### Conflict of Interest

The authors declare that the research was conducted in the absence of any commercial or financial relationships that could be construed as a potential conflict of interest.

## Abbreviations

IUI: Intrauterine Infection/Inflammation
TLR: Toll-like receptor
PAMP: Pathogen-associated molecular pattern
VEGF: Vascular endothelial growth factor
DAMPs: Damage-associated molecular patterns
MMPs: matrix metalloproteinases.
